# Veteran Experiences With an mHealth App to Support Measurement-Based Mental Health Care: Results From a Mixed Methods Evaluation

**DOI:** 10.2196/54007

**Published:** 2024-05-10

**Authors:** Robin T Higashi, Bella Etingen, Eric Richardson, Jennifer Palmer, Mark S Zocchi, Felicia R Bixler, Bridget Smith, Nicholas McMahon, Kathleen L Frisbee, John C Fortney, Carolyn Turvey, Jennifer Evans, Timothy P Hogan

**Affiliations:** 1 Peter O’Donnell Jr School of Public Health University of Texas Southwestern Medical Center Dallas, TX United States; 2 eHealth Partnered Evaluation Initiative Veterans Affairs Bedford Healthcare System Bedford, MA United States; 3 Research and Development Service Dallas Veterans Affairs Medical Center Dallas, TX United States; 4 Center for Healthcare Organization and Implementation Research (CHOIR) Veterans Affairs Boston Healthcare System Boston, MA United States; 5 Department of Psychiatry Harvard Medical School Boston, MA United States; 6 Section of General Internal Medicine Boston University Chobanian & Avedisian School of Medicine Boston, MA United States; 7 Center for Healthcare Organization and Implementation Research (CHOIR) Veterans Affairs Bedford Healthcare System Bedford, MA United States; 8 Center of Innovation for Complex Chronic Healthcare (CINCCH) Hines Veterans Affairs Hospital Hines, IL United States; 9 Northwestern University Feinberg School of Medicine Chicago, IL United States; 10 Office of Connected Care Veterans Health Administration US Department of Veterans Affairs Washington, DC United States; 11 Center of Innovation for Veteran-Centered and Value-Driven Care Veterans Affairs Puget Sound Health Care System Seattle, WA United States; 12 Division of Population Health Department of Psychiatry and Behavioral Sciences University of Washington Seattle, WA United States; 13 Center for Access & Delivery Research and Evaluation Iowa City Veterans Affairs Health Care System Iowa City, IA United States; 14 Office of Rural Health Veterans Rural Health Resource Center – Iowa City Iowa City Veterans Affairs Health Care System Iowa City, IA United States; 15 Department of Psychiatry University of Iowa Iowa City, IA United States; 16 Office of Mental Health and Suicide Prevention US Department of Veterans Affairs Washington, DC United States

**Keywords:** measurement-based care, mobile health app, mental health, veteran, mHealth, support, mixed-methods evaluation, digital health

## Abstract

**Background:**

Mental health conditions are highly prevalent among US veterans. The Veterans Health Administration (VHA) is committed to enhancing mental health care through the integration of measurement-based care (MBC) practices, guided by its Collect-Share-Act model. Incorporating the use of remote mobile apps may further support the implementation of MBC for mental health care.

**Objective:**

This study aims to evaluate veteran experiences with Mental Health Checkup (MHC), a VHA mobile app to support remote MBC for mental health.

**Methods:**

Our mixed methods sequential explanatory evaluation encompassed mailed surveys with veterans who used MHC and follow-up semistructured interviews with a subset of survey respondents. We analyzed survey data using descriptive statistics. We then compared responses between veterans who indicated having used MHC for ≥3 versus <3 months using *χ*^2^ tests. We analyzed interview data using thematic analysis.

**Results:**

We received 533 surveys (533/2631, for a 20% response rate) and completed 20 interviews. Findings from these data supported one another and highlighted 4 key themes. (1) The MHC app had positive impacts on care processes for veterans: a majority of MHC users overall, and a greater proportion who had used MHC for ≥3 months (versus <3 months), agreed or strongly agreed that using MHC helped them be more engaged in their health and health care (169/262, 65%), make decisions about their treatment (157/262, 60%), and set goals related to their health and health care (156/262, 60%). Similarly, interviewees described that visualizing progress through graphs of their assessment data over time motivated them to continue therapy and increased self-awareness. (2) A majority of respondents overall, and a greater proportion who had used MHC for ≥3 months (versus <3 months), agreed/strongly agreed that using MHC enhanced their communication (112/164, 68% versus 51/98, 52%; *P*=.009) and rapport (95/164, 58% versus 42/98, 43%; *P*=.02) with their VHA providers. Likewise, interviewees described how MHC helped focus therapy time and facilitated trust. (3) However, veterans also endorsed some challenges using MHC. Among respondents overall, these included difficulty understanding graphs of their assessment data (102/245, 42%), not receiving enough training on the app (73/259, 28%), and not being able to change responses to assessment questions (72/256, 28%). (4) Interviewees offered suggestions for improving the app (eg, facilitating ease of log-in, offering additional reminder features) and for increasing adoption (eg, marketing the app and its potential advantages for veterans receiving mental health care).

**Conclusions:**

Although experiences with the MHC app varied, veterans were positive overall about its use. Veterans described associations between the use of MHC and engagement in their own care, self-management, and interactions with their VHA mental health providers. Findings support the potential of MHC as a technology capable of supporting the VHA’s Collect-Share-Act model of MBC.

## Introduction

Veterans are impacted by mental health conditions at disproportionately higher rates in comparison to nonveteran US adults [1]. Evidence further suggests that veterans are motivated to use technology to enhance their health [2,3], and respond positively to digital health interventions, including those that address their mental health needs [4-6]. Currently, there are at least a dozen mental and behavioral health apps on VAMobile, the Veterans Health Administration (VHA) mobile health app store, including those targeting support for veterans with depression, posttraumatic stress disorder (PTSD), anxiety, and insomnia. Studies evaluating app effectiveness have shown positive outcomes; for example, the VHA’s PTSD Coach and Virtual Hope Box, two of their most popular mental health apps, showed significant improvements in symptom reduction and coping ability among app users versus control patients [7-9]. Thus, incorporating the use of mobile apps may further support the implementation of measurement-based care (MBC) for mental health care.

MBC is the systematic evaluation of patient symptoms using data collected from patients, including patient-reported outcome (PRO) measures, to inform health care and treatment decision-making [10,11]. In the context of mental health care, the integration of MBC practices has been demonstrated to enhance patient satisfaction and mental health outcomes [12-14]. The American Psychological Association endorses routine monitoring of symptoms, such as the use of PROs in MBC, as part of the delivery of evidence-based psychological practice [15].

The VHA, the largest integrated health care system in the United States [16], provides comprehensive mental health services across its nationwide system of care. The VHA launched its own MBC initiative in 2015 [17]. The VHA’s MBC initiative espouses a Collect-Share-Act model, in which veteran PRO data are collected, shared between veterans and VHA care team members, and used to inform treatment and facilitate shared decision-making [18]. The VHA’s Collect-Share-Act model was developed in light of research indicating that simply collecting assessment data from patients without facilitating patient-provider discussion of assessment results often fails to improve PROs such as symptom severity and feelings of well-being [19]. In addition to its Collect-Share-Act model, the VHA has also invested in various technology platforms intended to support remote measurement-based care (R-MBC).

R-MBC refers to the collection of PROs outside of the clinical encounter, typically with support from technologies including but not limited to secure messaging through a patient portal or mobile health apps. R-MBC is envisioned as a means to extend the reach of MBC by remotely tracking, monitoring, and asynchronously communicating symptoms to providers and facilitating patient self-management. Asynchronous R-MBC addresses a key barrier to MBC, disruption of clinic visit workflow due to the time it takes to administer and score assessments. Although recent systematic reviews have described mixed impacts of R-MBC [20], previous trials of interventions that have included R-MBC as part of effective mental health treatment have demonstrated improvements in clinical outcomes (eg, response to therapy and depression remission, adherence to medication, and satisfaction with depression treatment) in comparison to usual care [21,22].

Mental Health Checkup (MHC) is a mobile health app that was developed by the VHA Office of Connected Care to support R-MBC in VHA mental health services. Veterans can log on to MHC on the web using approved log-in credentials. Using MHC, veterans are assigned assessments by their VHA mental health providers that they may complete outside of a clinical encounter on a smartphone, tablet, or personal computer at intervals specified and assigned by providers. When a provider assigns assessments to a veteran in MHC, they receive an auto-generated email with a link that they can follow to complete the assessments, and reminders from the app when they have assigned assessments that they need to but have not yet completed. The app also includes a data visualization feature, wherein veterans can view graphs of their assessment results over time to monitor symptoms and progress. Additional app features allow mental health providers to receive alerts on veteran assessment scores, as well as notifications when veterans have completed assessments in between their appointments.

Despite the potential of technologies like the MHC app to facilitate R-MBC, few studies to date have described patient experiences using them. We conducted a mixed methods evaluation to assess veteran perspectives on and experiences with using the VHA MHC app, including their perspectives on its usability and applicability to their mental health care, and suggestions for enhancing the app.

## Methods

### Design

Our evaluation used a sequential-explanatory design comprising mailed surveys and semistructured telephone interviews with a national sample of veterans who were users of VHA’s MHC app. “Users” were defined as veterans who had ≥2 unique log-ins to the MHC app to account for those veterans who may have logged in once to explore the app but not used it.

### Recruitment and Data Collection

#### Surveys

We developed a survey with input from VHA operational leadership and mental health providers, including some involved in facilitating VHA’s MBC initiative. The survey was designed to address the potential impacts of the MHC app on the facets of MBC that comprise VHA’s Collect, Share, Act model of MBC, as well as other important constructs highlighted by stakeholders. Survey topics included frequency of use, preferences, barriers, and satisfaction with using the MHC app. A veteran member of our evaluation team reviewed survey question items for clarity and readability. We initiated the first survey mailing to 2690 veterans in June 2021. We initiated a follow-up mailing to nonresponders in July 2021 to facilitate response. Included in the survey mailings were a cover letter and a postage-paid reply envelope. We gave veterans the option to complete surveys by phone as preferred. Survey respondents were subsequently mailed a US $10 CVS (CVS Pharmacy, Inc) gift card in appreciation of their time.

#### Semistructured Interviews

Consistent with data integration through “building” in mixed methods [23], we recruited a subset of survey respondents to participate in follow-up semistructured telephone interviews intended to elaborate on survey responses. We purposefully stratified the interview sample to reflect variation in the following survey responses: app use and experiences with the app, including number of unique log-ins, app satisfaction, types of assessments completed in the app, age, and gender. We developed a semistructured interview guide with feedback from VHA operational leadership and VHA mental health providers and questions that broadly mapped to the domains of the well-established Non-Adoption, Abandonment, and Challenges to the Scaleup, Spread, and Sustainability (NASSS) of Health and Care Technologies Framework [24].

Interviews assessed veteran experiences with completing assessments in the app and using the app’s other features, as well as the perceived impacts of app use on a veteran’s care. Specifically, we asked veterans to describe (1) the condition for which they were using the MHC app, and the mental health care that they receive; (2) their previous experiences with MBC and how using MHC compares (eg, Can you please tell me about if and how you completed VA mental health care assessments before you began using MHC?); (3) their current experiences with R-MBC (eg, Are you still currently using the MHC app to complete assessments assigned to you by your VA mental health providers?); (4) their experience learning to use and using the MHC app (eg, What challenges have you encountered using MHC? What do you think is valuable about MHC?); (5) their perceived impact of MHC use on their mental health care, self-management, and outcomes (eg, How has completing assessments through MHC impacted your experiences with your VA mental health treatment?); and (6) their suggestions for improving MHC/R-MBC (eg, What changes could be made to make using the MHC app easier for veterans?).

Two evaluation team members with qualitative expertise (JP and FRB) conducted all interviews by telephone between August 2021 and September 2021, which coincided with the COVID-19 pandemic. Interviews lasted an average of 42 minutes (range 17-76 minutes). Each interview was audio recorded with the veteran’s permission and then professionally transcribed verbatim. Veterans who participated in an interview were mailed a US $20 CVS gift card in appreciation of their time.

### Data Analysis

#### Surveys

We characterized responses to the survey using descriptive statistics (frequencies and proportions) and compared survey responses between veterans who indicated on their survey that they used the app for ≥3 months with veterans who used the app for ˂3 months using the chi-square test statistic. Only veterans who indicated that they received and completed an assessment in MHC and also responded to the survey question regarding the duration of MHC use were included in the analyses. Stata (version 17; StataCorp LLC) was used for analysis of the survey data.

#### Semistructured Interviews

We analyzed interview data using thematic analysis facilitated by NVivo (version 12.0; QSR International). Two qualitatively trained investigators (RTH and JP) initially drafted a codebook using a deductively driven approach informed by topics in the interview guide. They jointly coded the first 25% of transcripts (5/20 interviews), iteratively refining the codes and definitions in an inductive manner. They then finalized the codebook and independently coded all the remaining transcripts. Any cross-analyst discrepancies in coding decisions were resolved through discussions in weekly meetings. We reviewed and synthesized findings in thematic reports, noting the frequency and emphasis of veteran comments, and selected exemplary quotes. These synthesized reports were then reviewed and discussed with the larger evaluation team for interpretation and integration with the survey findings.

We used a number of established methods [25] that strengthen the rigor and credibility of our findings, including using a sequential-explanatory design that compared quantitative and qualitative data, triangulating interpretations through discussions among a team of qualitatively trained doctoral-level analysts, and incorporating both confirmatory and contrasting cases of emergent themes.

### Ethical Considerations

All evaluation procedures were reviewed by relevant VHA institutional review boards and designated as program evaluation for quality improvement purposes, exempting it from further oversight (VHA Handbook 1058.05).

## Results

### Overview

We received 533 surveys (533/2631 surveys, with 59 surveys excluded from the denominator because they were returned due to incorrect addresses, for a 20% response rate), of which 271 respondents indicated they completed an MHC assessment. As noted above, veterans who also responded to the question about their duration of MHC use (n=268) were included in our analyses.

### Demographic and Veteran Characteristics

Veteran sociodemographic data are shown in Table 1. Survey respondents were predominantly male (167/259, 65%), White (183/266, 69%), and aged 55 years or younger (160/266, 60.2%). Veterans who had used MHC for ≥3 months did not differ in sociodemographic characteristics from veterans who used MHC for <3 months.

We also completed 20 semistructured interviews. Interviewed participants were mostly White (14/20, 70%), male (13/20, 65%), lived between 16 and 60 minutes from their nearest VHA facility (15/20, 75%), had completed a bachelor’s degree or higher (13/20, 65%), and were diverse in age (6/20, 30% aged ≤45 years; 9/20, 45% aged between 46 and 65 years; and 5/20, 25% aged 66 years and older). More than half (12/19, 63%) received between 90% and 100% of their VHA care remotely in the prior year, with 16% (n=3) receiving 0%-10% and 16% (n=3) receiving 75%-89% of VHA care remotely.

**Table 1:** Sociodemographic characteristics of surveyed Mental Health Checkup (MHC) app users by duration of use (n=268).

		Total, n (%)	Duration of MHC use		*P* value	
			Less than 3 months, n (%)	3 months or longer, n (%)		
**Age (years; n=266)**						.54
	<25	1 (0.4)	0 (0)	1 (0.6)		
	25-35	31 (11.7)	13 (13.1)	18 (10.8)		
	36-45	61 (22.9)	28 (28.3)	33 (19.8)		
	46-55	67 (25.2)	20 (20.2)	47 (28.1)		
	56-65	63 (23.7)	21 (21.2)	42 (25.1)		
	66-75	37 (13.9)	15 (15.2)	22 (13.2)		
	76 or older	6 (2.3)	2 (2)	4 (2.4)		
**Sex (n=259)**					.28	
	Male	167 (64.5)	61 (61.6)	106 (66.3)		
	Female	88 (34)	35 (35.4)	53 (33.1)		
	Decline to answer	4 (1.5)	3 (3)	1 (0.6)		
**Race (n=266)**						
	American Indian or Alaskan Native	11 (4.1)	3 (3)	8 (4.8)	.49	
	Asian	11 (4.1)	3 (3)	8 (4.8)	.49	
	Black or African American	48 (18)	19 (19.2)	29 (17.4)	.71	
	Native Hawaiian or other Pacific Islander	6 (2.3)	2 (2)	4 (2.4)	.84	
	White	183 (68.8)	65 (65.7)	118 (70.7)	.40	
	Other	16 (6)	8 (8.1)	8 (4.8)	.28	
	Decline to answer	14 (5.3)	7 (7.1)	7 (4.2)	.31	
**Ethnicity (n=263)**						.70
	Yes, Hispanic or Latino	39 (14.8)	14 (14.1)	25 (15.2)		
	No, not Hispanic or Latino	207 (78.7)	77 (77.8)	130 (79.3)		
	Decline to answer	17 (6.5)	8 (8.1)	9 (5.5)		
**Relationship status (n=266)**						.93
	Married or civil union	154 (57.9)	61 (61.6)	93 (55.7)		
	Engaged or in a relationship	17 (6.4)	5 (5.1)	12 (7.2)		
	Single, never married	35 (13.2)	12 (12.1)	23 (13.8)		
	Separated	11 (4.1)	4 (4)	7 (4.2)		
	Divorced	40 (15)	15 (15.2)	25 (15)		
	Widowed	5 (1.9)	1 (1)	4 (2.4)		
	Decline to answer	4 (1.5)	1 (1)	3 (1.8)		
**Education (n=266)**						.29
	Some high school	1 (0.4)	1 (1)	0 (0)		
	High school graduate or equivalent (eg, GED^a^)	21 (7.9)	9 (9.1)	12 (7.2)		
	Some college or vocational School	72 (27.1)	26 (26.3)	46 (27.5)		
	Associate degree	36 (13.5)	8 (8.1)	28 (16.8)		
	Bachelor degree	62 (23.3)	28 (28.3)	34 (20.4)		
	Master degree	59 (22.2)	23 (23.2)	36 (21.6)		
	Professional school degree (eg, MD, DDC, and JD)	6 (2.3)	2 (2)	4 (2.4)		
	Other doctoral degree (eg, PhD and EdD)	4 (1.5)	0 (0)	4 (2.4)		
	Decline to answer	5 (1.9)	2 (2)	3 (1.8)		
**Travel time to VHA^b^ (n=265)**						.73
	<15 minutes	31 (11.7)	11 (11.2)	20 (12)		
	16-30 minutes	85 (32.1)	29 (29.6)	56 (33.5)		
	31-60 minutes	102 (38.5)	43 (43.9)	59 (35.3)		
	61-120 minutes	38 (14.3)	12 (12.2)	26 (15.6)		
	>120 minutes	9 (3.4)	3 (3.1)	6 (3.6)		
**Proportion of VHA care that was received remotely in the prior year? (n=242)**						.14
	0-10	11 (4.5)	2 (2.2)	9 (6)		
	11-49	13 (5.4)	2 (2.2)	11 (7.3)		
	50-74	41 (16.9)	20 (22)	21 (13.9)		
	75-89	43 (17.8)	15 (16.5)	28 (18.5)		
	90-100	134 (55.4)	52 (57.1)	82 (54.3)		

^a^GED: General Education Development.

^b^VHA: Veterans Health Administration.

### Key Findings

#### Overview

Findings from the integrated quantitative and qualitative data sets supported one another and highlighted 4 key themes [23]. Specifically, (1) the use of the MHC app had positive impacts on care processes for veterans, including increased engagement in mental health care and self-management and (2) enhanced veteran-provider communication and rapport. However, (3) veterans also reported a variety of challenges using MHC. Last, (4) interviewees offered suggestions for improving the app’s future functionality and adoption.

#### Theme #1: Enhancing Self-Management and Engagement in Mental Health Care

Veterans reported completing a variety of assessments in the MHC app: PTSD (209/267, 78%), depression (191/267, 72%), and anxiety (180/267, 67%) were most common; others included assessments related to sleeping habits (125/267, 47%), quality of life (102/267, 38%), alcohol use (75/267, 28%), and physical health (68/267, 26%). Approximately two-thirds (169/268) of survey respondents indicated that they had been using MHC for ≥3 months. Veterans using the app for ≥3 months rated their overall satisfaction with the app similarly to those who used MHC for <3 months. However, a greater proportion of veterans using the app for ≥3 months endorsed several benefits of using the app (Table 2). A greater proportion of veterans who used MHC for ≥3 months also reported agreement that MHC helped to improve their VHA mental health care when compared with those who used it for <3 months (101/163, 62% versus 44/98, 45% agreed or strongly agreed, respectively; *P*=.007).

The MHC app offers veterans the ability to view their assessment data in graphs over time. While only 39% (n=104) of survey respondents reported viewing graphs of their assessment results, those who did described how the visualizations helped them gauge personal progress.

We were able to use that graph also in talking about, like, “Hey, during this time frame we were really peaking in your therapy.” You could see where your severity was high and how it’s come down and leveled out. So, I think that was also a good visual to be able to use as well.

veteran 0522

For some, the visualizations of their assessment data were an encouraging factor to continue therapy.

I really wanted to give it an opportunity to see if I could make any progress because it’s been a challenge for so long. Then after four or five weeks we looked back at the scores and where I was and how far I had come. Even though it wasn’t by much, it was enough to give me more encouragement to stay in therapy to keep me going, because she [provider] would go back, and she would review the graph and you could see in the graph where you’re making progress.

Veteran 0367

Seeing their assessment scores also enhanced some veterans’ self-awareness. As one veteran said, “it lets you know whether you’re in the ‘green level’ or whether you need to seek more help.” Others remarked:

It really helps me. It sure helps my therapist to see those numbers, but it helps me go, “Gosh, yeah, we’re still here? I’m still stuck?” It definitely helps you if you’re stuck to realize that you’re still stuck.

Veteran 0230

I can look at past surveys [ie, self-assessments] and, noticing that I have higher scores on some weeks, I think back, okay, what was going on during that week that may have caused my scores to be higher? Then I can go back and reflect on those and say, “Okay, what could have been done differently, if anything?”

Veteran 0367

**Table 2:** Impact of Mental Health Checkup (MHC) app on veteran self-management and engagement (n=268).

		Total, n (%)	Duration of MHC use			*P* value	
			Less than 3 months, n (%)	3 months or longer, n (%)			
**Overall satisfaction with MHC** **(n=262)**							.22
	Satisfied/strongly satisfied	189 (72.1)	65 (67.7)	124 (74.7)			
	Neutral/somewhat dissatisfied/very dissatisfied	73 (27.9)	31 (32.3)	42 (25.3)			
**Using MHC has helped with improving health and health care engagement** **(n=262)**							.03
	Agree/strongly agree	169 (64.5)	55 (56.1)	114 (69.5)			
	Neutral/disagree/strongly disagree	93 (35.5)	43 (43.9)	50 (30.5)			
**Understanding of one’s health condition** **(n=262)**							.02
	Agree/strongly agree	171 (65.3)	55 (56.1)	116 (70.7)			
	Neutral/disagree/strongly disagree	91 (34.7)	43 (43.9)	48 (29.3)			
**Health management** **(n=262)**							.01
	Agree/strongly agree	154 (58.8)	48 (49)	106 (64.6)			
	Neutral/disagree/strongly disagree	108 (41.2)	50 (51)	58 (35.4)			
**Health-related goal-setting (n=262)**							.06
	Agree/strongly agree	156 (59.5)	51 (52)	105 (64)			
	Neutral/disagree/strongly disagree	106 (40.5)	47 (48)	59 (36)			
**Health-related goal-achievement (n=262)**							.03
	Agree/strongly agree	133 (50.8)	41 (41.8)	92 (56.1)			
	Neutral/disagree/strongly disagree	129 (49.2)	57 (58.2)	72 (43.9)			
**Completion of assessments (n=262)**							.50
	Agree/strongly agree	196 (74.8)	71 (72.4)	125 (76.2)			
	Neutral/disagree/strongly disagree	66 (25.2)	27 (27.6)	39 (23.8)			
**More frequently completing assessments (n=262)**							.10
	Agree/strongly agree	174 (66.4)	59 (60.2)	115 (70.1)			
	Neutral/disagree/strongly disagree	88 (33.6)	39 (39.8)	49 (29.9)			
**Improving VHA^a^ mental health care (n=261)**							.007
	Agree/strongly agree	145 (55.6)	44 (44.9)	101 (62)			
	Neutral/disagree/strongly disagree	116 (44.4)	54 (55.1)	62 (38)			

^a^VHA: Veterans Health Administration.

#### Theme #2: Enhancing Veteran-Provider Communication and Rapport

A greater proportion of veterans who had used MHC for ≥3 months (versus <3 months) agreed that using the app helped them better communicate (112/164, 68% versus 51/98, 52% agreed/strongly agreed; *P*=.009) and improve rapport (95/164, 58% versus 42/98, 43% agreed/strongly agreed; *P*=.02) with their VHA providers (Table 3).

Consistent with the survey findings, in interviews, veterans reported that using MHC facilitated communication with their providers, particularly initiating or directing therapy conversations. One veteran reported that MHC allowed their provider to “open the book on me.” Others said:

It kind of gives them [providers] a ballpark idea and it gives them an opportunity of where they may start in asking questions and maybe where to open up the conversation, especially since I’m very private and unless I come to the table with a specific topic, I may not be one to open very easily. So, he [provider] may need some kind of information to help him pull information out of me. And this [sharing the Mental Health Checkup scores] can help him do that.

Veteran 0522

It makes you open up because once you accept...this is what’s happening to you, this is the way you’ve been feeling, it’s easier when she [provider] has it in front of her and she’s talking to me. That app helped me to face exactly what she was trying to get me to face because it was there – it was happening.

Veteran 0066

**Table 3:** Impact of Mental Health Checkup (MHC) on veteran-provider interactions (n=268).

			Total, n (%)		Duration of MHC use					*P* value
					Less than 3 months, n (%)		3 months or longer, n (%)			
**Using MHC has helped with communicating with VHA^a^ providers (n=262)**										.009
	Agree/strongly agree	163 (62.2)		51 (52)		112 (68.3)				
	Neutral/disagree/strongly disagree	99 (37.8)		47 (48)		52 (31.7)				
**VHA providers understanding how veterans are doing in between visits (n=262)**										.51
	Agree/strongly agree	201 (76.7)		73 (74.5)		128 (78)				
	Neutral/disagree/strongly disagree	61 (23.3)		25 (25.5)		36 (22)				
**Improving rapport with veterans’ providers (n=262)**										.02
	Agree/strongly agree	137 (52.3)		42 (42.9)		95 (57.9)				
	Neutral/disagree/strongly disagree	125 (47.7)		56 (57.1)		69 (42.1)				
**Making treatment decisions with veterans’ VHA providers (n=262)**										.14
	Agree/strongly agree	157 (59.9)		53 (54.1)		104 (63.4)				
	Neutral/disagree/strongly disagree	105 (40.1)		45 (45.9)		60 (36.6)				

^a^VHA: Veterans Health Administration.

Other veterans explained that sharing assessment scores from MHC improved communication with their providers because it helped them focus and structure their therapy time. As one veteran stated, the assessment scores help his provider “work more on what she knows I’m in need of and what we’re supposed to be going into.” Others said:

We didn’t have to waste time at the beginning of my appointment going over questions about “How are you doing?”. It was already done, so you could get into things, where let’s say you said that you’re really depressed, you know, you don’t have to go into asking that question to begin with.

Veteran 0501

I guess it gave me the feeling that it made it more structured, which is something I like. I like structure.

veteran 0505

Importantly, discussing assessment findings also facilitated rapport and trust.

Psychologists in the past were just like, “Oh yeah, just fill this out” and, you know, they never talked about it with me. I didn’t know what I was filling out.... This [provider] actually does care. That kind of gave me a breath of fresh air, like “Oh okay, he takes his job very seriously and cares about me and my health”.... Now we know what the underlining issue is, and I think, without the app, we probably never would have done that.

Veteran 0077

It has made me more comfortable and trusting, where we [veteran and provider] decide together what’s needed, for adjustment or to stay the same. So, it’s like I don’t have that stress of worrying about what’s gonna happen to me because I know whatever we come up with.... It’s gonna be something that is really gonna benefit me and help me.

Veteran 0111

#### Theme #3: Challenges Using MHC

While veterans described how the use of the MHC app had positive impacts on their self-management and engagement in care, as well as their interactions with their providers, both the survey and interview findings indicated that MHC posed several challenges for veterans.

Survey findings indicated that veterans who used MHC for <3 months reported almost all challenges at a similar frequency as veterans who used the app for ≥3 months (Table 4). However, a greater proportion of veterans who had used the MHC app for <3 months reported that not being able to answer assessment questions as well as they could when they were at a VHA facility was a somewhat serious or serious challenge (32/98, 33% versus 30/166, 18%; *P*=.007). Despite the challenges reported, 78% (n=207) of veterans expressed that they were comfortable using the app, and over 41% (n=108) described not having trouble using the MHC app. When veterans encountered trouble using MHC (N=154), the most frequently reported actions they took were securely messaging (84/154, 55%) or calling their VHA providers (57/154, 37%), and to a lesser extent, calling the VHA Help Desk for technical support (29/154, 19%).

**Table 4:** Veteran-reported challenges when using the Mental Health Checkup (MHC) app by duration of use (n=268).

		Total, n (%)	Duration of MHC use			*P* value	
			Less than 3 months, n (%)	3 months or longer, n (%)			
**Unable to access assessments (n=264)**							.63
	Somewhat/serious challenge	69 (26.1)	27 (27.8)	42 (25.1)			
	Not a challenge	195 (73.9)	70 (72.2)	125 (74.9)			
**All needed assessments not in MHC (n=248)**							.46
	Somewhat/serious challenge	51 (20.6)	21 (23.1)	30 (19.1)			
	Not a challenge	197 (79.4)	70 (76.9)	127 (80.9)			
**Unable to correct erroneous responses (n=256)**							.25
	Somewhat/serious challenge	72 (28.1)	31 (32.3)	41 (25.6)			
	Not a challenge	184 (71.9)	65 (67.7)	119 (74.4)			
**Unable to save answers (n=257)**							.25
	Somewhat/serious challenge	52 (20.2)	23 (24)	29 (18)			
	Not a challenge	205 (79.8)	73 (76)	132 (82)			
**Difficulty understanding graphs of assessment scores (n=245)**							.11
	Somewhat/serious challenge	102 (41.6)	43 (48.3)	59 (37.8)			
	Not a challenge	143 (58.4)	46 (51.7)	97 (62.2)			
**Inability of providers to see entered answers (n=246)**							.35
	Somewhat/serious challenge	39 (15.9)	17 (18.7)	22 (14.2)			
	Not a challenge	207 (84.1)	74 (81.3)	133 (85.8)			
**Providers and veterans do not talk about assessment scores (n=260)**							.97
	Somewhat/serious challenge	70 (26.9)	26 (26.8)	44 (27)			
	Not a challenge	190 (73.1)	71 (73.2)	119 (73)			
**Providers do not use assessment scores to inform my care (n=247)**							.60
	Somewhat/serious challenge	63 (25.5)	26 (27.4)	37 (24.3)			
	Not a challenge	184 (74.5)	69 (72.6)	115 (75.7)			
**Inability to answer questions as well as when at the VHA^a^ (n=264)**							.007
	Somewhat/serious challenge	62 (23.5)	32 (32.7)	30 (18.1)			
	Not a challenge	202 (76.5)	66 (67.3)	136 (81.9)			
**Lacking a private place to complete assessments (n=263)**							.97
	Somewhat/serious challenge	19 (7.2)	7 (7.1)	12 (7.3)			
	Not a challenge	244 (92.8)	91 (92.9)	153 (92.7)			
**Insufficient training about MHC (n=259)**							.64
	Somewhat/serious challenge	73 (28.2)	29 (29.9)	44 (27.2)			
	Not a challenge	186 (71.8)	68 (70.1)	118 (72.8)			
**Providers did not explain how they wanted MHC used (n=258)**							.18
	Somewhat/serious challenge	65 (25.2)	29 (29.9)	36 (22.4)			
	Not a challenge	193 (74.8)	68 (70.1)	125 (77.6)			
**Not enough help available for troubleshooting with MHC (n=254)**							.56
	Somewhat/serious challenge	64 (25.2)	22 (23.2)	42 (26.4)			
	Not a challenge	190 (74.8)	73 (76.8)	117 (73.6)			
**Limited veteran access to a mobile/computing device (n=263)**							.50
	Somewhat/serious challenge	23 (8.7)	7 (7.2)	16 (9.6)			
	Not a challenge	240 (91.3)	90 (92.8)	150 (90.4)			
**Limited veteran access to the Internet or sufficient Wi-Fi capacity (n=264)**							.39
	Somewhat/serious challenge	33 (12.5)	10 (10.2)	23 (13.9)			
	Not a challenge	231 (87.5)	88 (89.8)	143 (86.1)			
**Concerns about privacy and security of responses entered into MHC (n=263)**							.49
	Somewhat/serious challenge	66 (25.1)	22 (22.7)	44 (26.5)			
	Not a challenge	197 (74.9)	75 (77.3)	122 (73.5)			

^a^VHA: Veterans Health Administration.

Veterans described similar challenges within the follow-up semistructured interviews. Notably, many of the challenges they discussed were not unique to the MHC app; rather, they pertained to uncertainty about the purpose of MBC; emotional burden related to completing assessments; and challenges related to PRO assessments more generally.

One veteran, confused about the impact of his scores on his overall mental health care, noted his uncertainty about the purpose of measurement: “I wasn’t sure what the results or effect was gonna be.”

Some veterans also reported emotional burdens related to completing assessments. For example, completing assessments could be emotionally triggering.

I hate doing them because it just brings up, you know, everything I’m trying to avoid.... It just reminds me like I’m not getting any better or still have these issues.

Veteran 0077

Emotional burden was also experienced when a veteran felt anxiety related to the assessments: feeling “pressure” about not scoring well or “failing the program” if they did not score well and the implications this might have for their provider:

...when you don’t see progress...it almost like nullifies the work you’re doing, right, so you’re like, oh,.... I’m failing at this program...and I’m also failing the provider that I’m working with because now he can’t show his provider or his boss that he’s being successful with me, so that adds another level – another layer of – of something to work through. ... It’s another layer of pressure. ... it was always hanging out there, right, you know, like almost a sense of dread, like, okay, I gotta show some dots moving up, you know?

Veteran 0041

Last, some veterans experienced challenges with PRO assessments themselves. First, a veteran found assessing their emotional status “... hard to quantify.... There’s no definition of what severe means... ” (Veteran 0041)

Assessments could also be challenging given competing demands on a veteran’s attention and time:

I think the problem is more me than you guys or your app, you know, in my lack of mindfulness and my forgetfulness and my just lifestyle don’t make it super-easy to carve out time to give it the attention that it deserves.

Veteran 0047

Another veteran pointed out how completing the same type of assessment over time was “tedious*...*just because it’s the same questions every time.” (Veteran 0087)

Veterans found inadequate instructions about how to use the app to be a further challenge in completing assessments.

#### Theme #4: Suggestions for Improving MHC

In addition to sharing comments about the challenges they faced, interviews also provided an ideal opportunity for veterans to share their thoughts about how to overcome some of the aforementioned challenges and how to improve the functionality of the MHC app. Technical suggestions included reducing the number of steps to log into MHC to just a username and password, and including a reminder function (eg, an alarm) or a link to one’s electronic calendar to support the completion of assessments and, by extension, sustained engagement. Other suggestions pertained to the assessments themselves. veterans described the potential utility of defining scale terms (eg, does “frequently” mean 3 times/week or 5 times/week?), reordering assessment items so they do not feel redundant, and reducing the time frames over which veterans are asked to reflect upon and recall the symptoms they are experiencing. Veterans also wanted providers to talk to them about how assessments “fit into the big picture” of their mental health care. Finally, some veterans offered their thoughts about ways to support the broader implementation of the MHC app, including marketing campaigns within and beyond clinical settings, providing individual training opportunities and further technical support, and ensuring awareness of the MHC app and its consistent use in practice among VHA mental health providers.

## Discussion

### Principal Findings

Survey and interview findings revealed that, although veteran experiences varied, use of MHC, an app designed to support R-MBC for mental health care in the VHA, appeared to be associated with several self-reported increases in mental health care benefits, particularly for veterans who used the app for ≥3 months. Compared with veterans using MHC for <3 months, veterans who used MHC for ≥3 months were more likely to report that MHC helped them be more engaged in treatment, manage their health, and communicate with their VHA providers. These findings were echoed among assertions from interviewed veterans. At the same time, veterans also identified several usability challenges and other issues related to MBC more generally and the use of technologies to support R-MBC, as well as opportunities to improve upon MHC specifically.

These findings extend previous work that has shown that MBC improves communication between providers and patients [11,26,27]. While veterans in our sample also reported improved communication with providers, a unique feature of our findings is that veterans further expressed a sense of enhanced rapport, engagement, and self-management of their conditions. The specific features offered by technology platforms that are intended to support R-MBC can also reflect different philosophies about the use of patient health information to support clinical care.

Previous literature has suggested that MBC approaches may be used to bolster patient self-management and engagement in care [28]. As noted above, and unlike some other existing R-MBC platforms, the MHC app offers veterans the ability to review their own assessment scores and to view graphical representations of them over time. Our results similarly suggest that these are meaningful features that help at least some veterans to be more self-aware, motivated to continue their treatment, and stay in touch with their own progress. It is possible that such features could facilitate veteran symptom self-management and engagement in mental health care, and perhaps should be included in other R-MBC technology platforms.

Additionally, whereas most studies to date have focused on provider perspectives toward R-MBC [29-31], ours is one of a few that has examined patient perspectives and experiences with the processes of R-MBC and the kinds of technologies that can be used to support them, an equally important factor for uptake and implementation [32,33]. Finally, our evaluation’s use of semistructured interviews enabled veterans to characterize the breadth of their experiences in their own words and to offer open-ended suggestions, rather than being restricted to choosing from among predetermined survey response options. Such feedback may be particularly helpful for enhancing MBC and R-MBC processes and the technologies used to support them, in that veterans have the opportunity to share, in narrative form, their thoughts on app enhancement strategies and ways to best incorporate veteran perspectives into MBC processes.

Our qualitative work yielded additional findings new to the field; that is, veterans offered valuable suggestions that could enhance the usability and future implementation of the MHC app as well as other possible R-MBC technology platforms among veterans and their VHA mental health providers. Suggestions included offering new ways to remind veterans to complete the assessments they have been assigned as well as offering support to help veterans understand the meaning of the graphical representations of their assessment results within the app. This may help veterans stay more engaged between the time they complete an assessment and their next appointment with their VHA mental health provider.

Challenges related to log-in validation and other usability issues underscored the potential benefit of having readily available training and technical support to enhance veteran uptake and continued use of the MHC app. It is also noteworthy that some veterans offered feedback that suggested they might benefit from a greater understanding of the goals of MBC more generally, which, in turn, might facilitate their completion of assessments and sharing of assessment data. Specifically, veterans suggested that it may be helpful for providers to explain what the PRO assessments they are asked to complete are intended to measure, why these assessments were assigned, and how the assessments are structured so that they are easier to navigate.

Taken together, our findings speak to the potential of the MHC app as a platform that can support VHA’s Collect-Share-Act model of MBC. Completing assessments through the MHC app (ie, the “collecting” stage of the Collect-Share-Act model) facilitated veterans’ abilities to express their emotions, which some described to be otherwise challenging. Additionally, the ability to view graphs of their assessment results helped some veterans gauge their personal progress and was a source of encouragement to continue with their therapy for others, as was the feedback that some veterans received from their mental health providers (ie, the “sharing” stage of Collect-Share-Act), especially for those who felt “stuck” or lacking in motivation. Veterans also felt that receiving feedback about their assessment scores played an important role in their willingness to use MHC given that, for some veterans, completing mental health assessments through a technology like the MHC app could be potentially upsetting. Finally, veterans expressed satisfaction with data being used during clinical appointments (ie, the “acting” stage of Collect-Share-Act) to inform treatment decisions such as how best to continue care. In sum, this evaluation of MHC underscored that each phase of the Collect-Share-Act model serves a critical role in supporting MBC for mental health care to help meet veteran mental health care needs.

### Limitations

Our surveys and interviews captured the self-reported behaviors and attitudes of veterans who have used the MHC app; thus, these methods may be affected by common problems of self-reported data including recall, desirability, and other types of bias. Because we conducted data collection during the COVID-19 pandemic, a large proportion (134/242, 55%) of the veterans in this evaluation reported that 90%-100% of their care was virtual over the year preceding participation. Attitudes toward the MHC app may have differed if this evaluation was conducted during a time when care at a distance using technology was not considered “standard” practice. It is also possible that interviews conducted via telephone may have restricted the incorporation of nonverbal cues that may have enriched our analysis. Additionally, because our survey and interview samples comprised veterans who had at least some preceding experience using the MHC app and, by extension, were established users of VHA mental health care, our findings may not translate to those veterans who are newer to VHA mental health services. Future work to evaluate the effectiveness of MHC should qualitatively assess the experiences of veterans who have used MHC for longer durations to better understand their perspectives toward the usability of the app, benefits, challenges, and suggestions for enhancements. Future work may also benefit from a more rigorous design, such as randomization and less reliance on self-reported data, to evaluate the relationships between MHC use and clinical outcomes. Finally, like the general population, digital disparities exist in the veteran population [34,35]; technical training and targeted assistance to older, rural-residing, and less technologically literate veterans will be key to the success of any R-MBC app, including MHC.

### Conclusions

Among a national sample of veterans, those who used the VHA’s MHC app for ≥3 months were more likely to report better engagement and self-management of their condition (ie, understanding their condition, managing their health, and achieving their health and health care goals), increased communication and rapport with their VHA providers, and improved aspects of their mental health care compared with veterans who used the app for <3 months. Despite some challenges with usability, the overwhelming majority reported that they were comfortable using the app. Suggestions from interviewed veterans included streamlining the log-in process, implementing enhanced training and technical assistance, reminder tools, orientation to assessment design, and enhanced marketing to veterans and providers. These findings suggest that MHC may be a valuable tool to enhance veteran mental health care in accordance with the VHA’s Collect-Share-Act model and should be further evaluated to assess its effectiveness in enhancing clinical outcomes.

## Abbreviations

MBC: measurement-based care
MHC: Mental Health Checkup
NASSS: Non-Adoption, Abandonment, and Challenges to the Scaleup, Spread, and Sustainability
PRO: patient-reported outcome
PTSD: posttraumatic stress disorder
R-MBC: remote measurement-based care
VHA: Veterans Health Administration
